# Tankyrase Promotes Aerobic Glycolysis and Proliferation of Ovarian Cancer through Activation of Wnt/*β*-Catenin Signaling

**DOI:** 10.1155/2019/2686340

**Published:** 2019-02-20

**Authors:** Hong-Yi Yang, Jin-Xing Shen, Yi Wang, Yu Liu, Dong-Yan Shen, Song Quan

**Affiliations:** ^1^Center for Reproductive Medicine, Department of Obstetrics and Gynecology, Nanfang Hospital, Southern Medical University, Guangzhou 510515, China; ^2^Department of Biobank, The First Affiliated Hospital, School of Medicine, Xiamen University, Xiamen 361003, China

## Abstract

Tankyrase (TNKS) plays important roles in the malignancy of several cancers such as human lung tumor, breast cancer, and hepatocellular cancer. However, its exact functions and molecular mechanisms in ovarian cancer remain unclear. In this study, we found that TNKS was aberrantly overexpressed in human ovarian cancer tissues and associated with poor patient prognosis. TNKS inhibition or knockdown not only reduced ovarian cancer cell proliferation, colony formation, migration, invasion, and tumorigenic potential in nude mice but also enhanced the drug susceptibility of ovarian cancer cells through arresting cell cycle and inducing apoptosis. These phenotypic changes correlated with downregulation of targets (Cyclin D1, MDR, and MMP-9) of Wnt/*β*-catenin signaling. Furthermore, downregulation of TNKS suppressed the glucose uptake, lactate excretion, and cellular ATP levels and increased cellular O_2_ consumption rates. Molecular mechanism studies revealed that TNKS promoted aerobic glycolysis at least in part due to upregulation of pyruvate carboxylase (PC) via activation of Wnt/*β*-catenin/snail signaling. In agreement with these findings, expression of TNKS is positively associated with snail and PC in clinical ovarian cancer samples. Our findings identified TNKS as an oncogenic regulator of ovarian cancer cells proliferation that promotes aerobic glycolysis via activation of Wnt/*β*-catenin signaling, indicating that the TNKS might serve as a potential molecular target for clinical therapy of Wnt/*β*-catenin dependent ovarian cancer.

## 1. Introduction

Ovarian cancer is the fourth leading cause of gynaecological malignancy around the world, and estimated 239,000 new patients are diagnosed with ovarian cancer each year [1, 2]. Currently, combination of debulking surgery and systemic chemotherapy is the traditional treatment of ovarian cancer [3]. However, about 70% ovarian cancer patients will relapse after chemotherapy due to drug resistance [4]. Therefore, it is urgent to address the mechanisms underlying ovarian cancer proliferation, metastasis, and chemoresistance for the development of novel therapeutic strategies.

Wnt signaling is essential during multiple embryonic developmental progresses and its aberrant activation plays a crucial role in the proliferation, differentiation, metastasis, and multidrug resistance of many human cancers, including ovarian cancer [5, 6]. Wnt signaling can be activated after the combination of Wnt ligands to cell surface receptors, leading to the stabilization of *β*-catenin, which activates the canonical Wnt pathway [7]. There are various downstream proteins and genes that participate in the Wnt/*β*-catenin pathway including Snail (*SNAIL*), Axin2 (*AXIN2*), and matrix metalloproteinases (*MMPs*). In addition, it is common that the related genes of Wnt/*β*-catenin pathway are mutated in ovarian cancer, suggesting that the Wnt/*β*-catenin pathway plays a significant role in the development of ovarian cancer [8].

Tankyrases (TNKS) are members of the poly(ADP-ribose) polymerase (PARP) family, including TNKS1/PARP5A and TNKS2/PARP5B [9]. Both TNKS1 and TNKS2 destabilize the levels of Axin, thereby reducing *β*-catenin degradation with consequent hyperactivation of Wnt/*β*-catenin signaling [10]. Since their discovery as telomere-associated proteins in human cells, TNKS have been linked to multitude cellular functions, such as mitotic progression, glucose metabolism, and possible proteasome regulation [11]. Reflecting the multitude of their target proteins and cellular functions, TNKS have been implied in a variety of diseases including cancer, including lung cancer [12], hepatocellular cancer [13], gastric cancer [14], breast cancer [10], and colon cancer [15]. As such, TNKS is promising candidate targets for anticancer molecular therapies. However, the expression profile of TNKS protein and its roles and mechanisms in ovarian cancer remain unknown.

Given the strong implication of in the molecular pathogenesis of ovarian cancer, we rationalized that TNKS may play important roles in the development of ovarian cancer. In the current study, we found elevated TNKS expression in a subset of human ovarian cancer. TNKS knockdown in ovarian cancer cells inhibited cell proliferation, cell metastatic abilities, and drug susceptibility. Moreover, TNKS could promote aerobic glycolysis by inducing pyruvate carboxylase (PC) expression through activation of Wnt/*β*-catenin/snail signaling. Our study demonstrated that TNKS inhibition might give a reliable therapeutic benefit for ovarian cancer.

## 2. Material and Methods

### 2.1. Reagents and Antibodies

XAV939, Wnt3a, taxane, cisplatin (CDDP), MTS, and Glucose Oxidation assay kit were purchased from Sigma-Aldrich (St. Louis, MO, USA). Fetal calf serum and RPMI-1640 medium were from Gibco (Grand Island, NY, USA). Lipofectamine 2000, goat anti-mouse and anti-rabbit secondary antibodies conjugated to HRP were from Invitrogen (Carlsbad, CA, USA). Monoclonal antibodies against GAPDH (sc-47724), GLUT1 (sc-377228), PDK1 (sc-293160), cyclin D (sc-70899), snail (sc-271977), G6PD (sc-373886), GCK (sc-17819), DLD (sc-271569), ALDOA (sc-377058), PGK1 (sc-130335), PC (sc-166649) and polyclonal antibody against MDR (sc-55510), and MMP-9 (sc-21733) were from Santa Cruz Biotechnology (Santa Cruz, CA, USA). Polyclonal antibodies against TNKS (ab227471), HK (ab150423), *β*-catenin (ab32572), and phosphorylated *β*-catenin (ab27798) were from Abcam Ltd. (Cambridge, United Kingdom). The polyvinylidene difluoride (PVDF) membrane and the annexin V fluorescein isothiocyanate (FITC)/propidium iodide (PI) double-staining apoptosis detection kit were obtained from Millipore (Billerica, MA, USA). The BioCoat Matrigel invasion chamber was from BD Biosciences, Inc. (Rockville, MD, USA). The Dual-Glo Luciferase Assay System kit was purchased from Promega (Madison, WI, USA) and the EliVision Plus kit was from Maixin Bio (Fuzhou, China). Lactate assay kit (BioVision) and an ATP Bioluminescence assay kit were purchased from Promega Thermo Scientific (Wilmington, DE, USA).

### 2.2. Patients and Tissue Samples

We followed the methods of our previous studies [16, 17]. Ovarian cancer and their adjacent noncancerous tissues were collected from 75 patients who underwent surgery at the First Affiliated Hospital of Xia-men University (Xiamen, China). Written informed consent was obtained from each patient. The study protocol conformed to the ethical guidelines of the 1975 Declaration of Helsinki, and it was approved by the Institute Research Ethics Committee of the First Affiliated Hospital of Xiamen University (Permit Number: 20150305-2, 5 March 2015). Fresh surgical samples from ovarian cancer tissues were collected between 2015 and 2017. All patients enrolled into the study did not receive preoperative treatment, such as radiation or chemotherapy. Metastatic tumors from other tissues were excluded from the study. The clinical data are shown in Table 1.

### 2.3. Immunohistochemistry

This method was performed as previously described [16, 17]. Paraffin-embedded human ovarian cancer tissue sections were pretreated with blocking buffer (5% normal goat serum in PBS) for 30 min at room temperature and then immunostained with antibody against TNKS (1:200) at 4°C overnight, followed by incubation with secondary antibody conjugated with HRP. Images were collected and analyzed using an inverted fluorescence microscope. The staining intensity of TNKS protein was categorized into four different grades according to their different positive rates. The staining intensity - and + were considered as low expression, and ++ and +++ were considered as high expression.

### 2.4. Cell Culture and Transfection

The three ovarian cell lines (ES-2, OVCAR-3, and A2780) and human normal ovarian epithelial (HNOE) cells were purchased from Cell Bank of the Chinese Academy of Sciences (Beijing, China). These four cell lines were cultured in RPMI-1640 supplemented with 10% FBS, 100 U/mL penicillin, and 100 U/mL streptomycin at 37°C in an atmosphere of 5% CO2. The OVCAR-3 cells were transfected with pll3.7 control vector (shCtrl) or pll3.7-TNKS vector (shTNKS) with Lipofectamine 2000 to obtain stably transfected cells.

### 2.5. Real-Time PCR

Total RNA was extracted using a Simple RNA Extraction kit (Tiangen, Beijing, China) according to the manufacturer's instructions as previously described [16, 17]. Reverse transcription was carried out using the SuperScript III First-Strand Synthesis System (In-vitrogen) for real-time RT-PCR. The RT-PCR for TNKS (Forward prime: GCTGCAGGTTTTGGAAGGAA; reverse prime: CCTGGCATTTGGATCAGCTC) was carried out in 96-well plates using a Light Cycler 480 (Roche Molecular Biochemicals, Mannheim, Germany). Relative quantification was analyzed by normalization to the amount of GAPDH (Forward prime: TCACCCACACTGTGCCCATCTACGA; reverse prime: CAGCGGAACCGCTCATTGCCAATGG), and relative expression level of genes was analyzed with the 2^−ΔΔCt^ method.

### 2.6. Western Blotting

Western blotting was carried out as previously described [16, 17]. Aliquots containing 15 g of protein underwent SDS-10% PAGE and then were transferred onto a PVDF membrane. The membrane was incubated with primary (1:1000) and secondary antibodies (1:5000), and the signal was finally visualized with an enhanced chemiluminescence (ECL) system. GAPDH was used as an internal control.

### 2.7. Cell Proliferation Assay

As previously described [16, 17], cells were seeded at 5 × 10^3^ into 96-well plates overnight or another time. After treatment with a variety of agents including taxane and CDDP, MTS (5 mg/mL) was added to each well. The plates were incubated at 37°C for 4 h and followed measured at 490 nm using a microplate reader (Model 680; Bio-Rad, Hercules, CA, USA).

### 2.8. Colony Formation Assay

As previously described [16, 17], five hundred cells were cultured in 6-well plates for 2 weeks. The colonies were fixed and stained with 0.005% crystal violet for 30 min and colonies > 100 *μ*m in diameter were counted.

### 2.9. Cell Migration and Invasion Assay

Cell migration was examined by wound-healing and transwell assays, and in vitro invasion was evaluated by using a Matrigel invasion chamber as previously described [17]. For wound-healing assays, cells were cultured in 24-well plates with 100% confluence and then wounded in a line across the slides with a micropipette tip. The medium was removed, and the monolayer was washed with PBS. Graphics were obtained by 0.05% crystal violet staining after 0 and 48 h of incubation. For transwell assays, 2.5 × 10^4^ cells were seeded per upper chamber in serum-free RPMI 1640, whereas the lower chambers were loaded with RPMI 1640 containing 5% FBS. After 24 h, the nonmigrating cells in the upper chambers were removed with a cotton swab, and cells migrating through the membrane to the underside of the membrane were stained with 0.05% crystal violet and counted. Cell invasion assays were performed similarly, but with Matrigel.

### 2.10. Flow Cytometry

Cell cycle and apoptosis analysis were performed by flow cytometry as previously described [16]. For cell cycle analysis, cells were synchronized to the G_0_/G_1_ phases by serum starvation and then cultured for 48 h. The cells were washed, harvested, fixed with ice-cold PBS in 70% ethanol, and stored at 4°C overnight. After centrifugation, the cells were resuspended in 100 g/ml RNase A at 37°C for 30 min and subsequently stained with 50 g/ml PI at 4°C for 30 min in the dark. The cells were analyzed with a FACScan flow cytometer (Becton Dickinson, Franklin Lakes, NJ, USA) at 488 nm. Apoptosis was evaluated using an annexin V-FITC/PI double-staining assay. The annexin V-FITC/PI double-staining assay was performed according to the manufacturer's instructions.

### 2.11. Analysis of Glucose Metabolism and Mitochondrial Activity

Cells were seeded onto 35 mm culture dishes, and after 6 h the culture medium was replaced with fresh complete medium and incubated for additional 48 h. The media were then collected for measurement of glucose and lactate concentration and cells harvested for protein lysates. Glucose levels were determined using a glucose assay kit. Lactate levels were determined using a lactate assay kit according to the manufacturer's instruction. Activity of mitochondrial respiratory chain complex I in cellular lysates was assessed using Complex I Enzyme activity microplate assay kit according to the manufacturer's protocol. All values were normalized on the basis of the Bradford protein assay.

### 2.12. Detection of ATP Level

Intracellular ATP level was assayed by a bioluminescence method with the ENLITEN ATP Assay System Bioluminescence Detection Kit according to the kit instructions. Cells were seeded in six-well plates for 24 h and then collected and homogenized with 500 ml of ice-cold homogenization buffer (0.25 M sucrose and 10 mM HEPES-NaOH (pH 7.4)). Equal volume of ice-cold 10% TCA (trichloroacetic acid) was added to homogenate and shaken for 20 s and then centrifuged at 10,000g for 10 min at 4°C. Supernatant (400 ml) was collected and added to 200 ml of Tris-acetate buffer (1 M, pH 7.75) for neutralization. The supernatant was diluted 50-fold with deionized water, and 20 ml of the diluted extract was used for ATP assay by a luminometer.

### 2.13. Dual-Luciferase Reporter Assay

OVCAR-3 cells (1.0 × 10^4^ cells/well) were seeded in 96-well plates for 24 h before transfection as previously described [16, 17]. Cells were transfected with shTNKS or shCtrl for 24 h. Then cells in each well were cotransfected with 100 ng pTOPFlash or pFOPFlash reporter plasmid and 20 ng Renilla luciferase expression vector using Lipofectamine 2000 for another 24 h and subsequently with Wnt3a for 6 h or XAV939 for 4 h. The indicated cells were analyzed for luciferase activity by using a Dual-Glo Luciferase Assay System (Promega), according to the manufacturer's instruction.

### 2.14. Tumor Xenografts

Four-week-old female BALB/c nude mice were used as previously described [17]. A total of 4 × 10^6^ cells were injected subcutaneously into the dorsal thighs of mice. The tumor volume was calculated every 3 days. Tumor volumes were determined according to the following formula: A × B^2^/2, where A is the largest diameter and B is the perpendicular diameter. Mice were sacrificed at day 21 after cell injection. All mice were kept under specific pathogen-free conditions at Xiamen University Laboratory Animal Center (Xiamen University, China) in accordance with institutional guidelines. This study was approved by the local Ethical Committee of the First Affiliated Hospital of Xiamen University (Permit Number: 20170407-12, 7 April 2017).

### 2.15. Statistical Analysis

Data were analyzed using GraphPad Prism software (San Diego, CA, USA). Data are obtained from at least three independent experiments and presented as the means ± SD. Student's t-test (two-tailed), one-way ANOVA, Fisher's exact test, and Pearson's r were used to compare data and to calculate their probability value (*P*). Multigroup comparisons of the means were carried out by one-way analysis of variance (ANOVA) test with post hoc contrasts by Student-Newman-Keuls test.* P* < 0.05 was considered statistically significant.

## 3. Results

### 3.1. Clinical Significance of TNKS1 Overexpression in Ovarian Cancer Tissues

**Figure 1:**
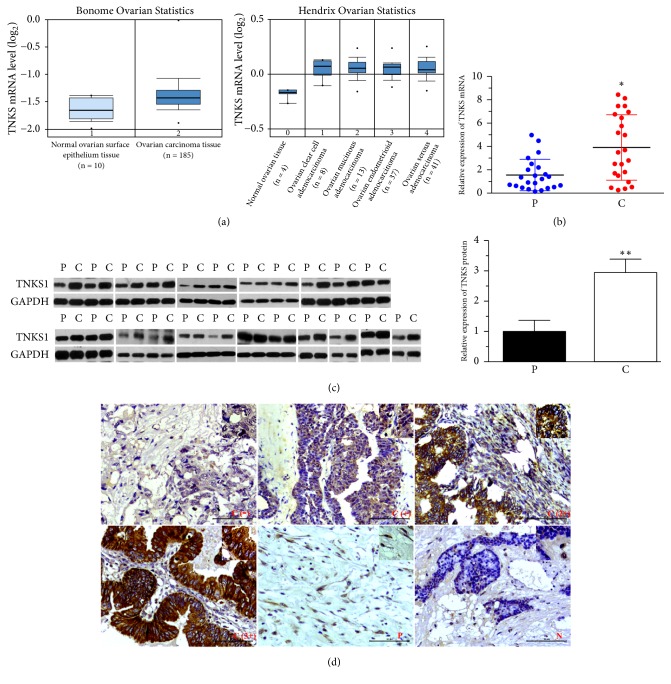
*Expression of TNKS in Clinical Ovarian Cancer Tissues*. (a) The levels of TNKS mRNA in ovarian cancer and normal ovarian surface epithelium tissues (left), and different histopathological types. The data are obtained from Bonome Ovarian Statistics and Hendrix Ovarian Statistics of Oncomine database, respectively. (b) Real-time PCR analysis of TNKS mRNA levels in clinical ovarian cancer and paracancerous tissues. (c) Western blotting analysis of TNKS1 protein levels in 23 clinical ovarian cancer and paracancerous tissues. GAPDH was used to indicate the amount of loading proteins. (d) Representative IHC of TNKS protein in C, P, and N tissues. Four different grades (-, +, 2+, and 3+) of staining intensity were according to their different positive rates of TNKS expression. Scale bar: 50 *μ*M. C: cancer; P: paracancerous; N: normal.

Gene expression data from Oncomine demonstrates that TNKS1 gene expression levels substantially higher in ovarian cancer tissues than in normal tissues (Figure 1(a)). Consistent with these biostatistics, elevated gene and protein expression levels in ovarian cancer tissues but decreased levels in the paired paracancerous samples (normal fallopian tube epithelium tissues) of TNKS were also observed in clinical samples (Figures 1(b) and 1(c)). In order to evaluate the significance of TNKS overexpression, immunohistochemistry (IHC) was used to analyze a series of ovarian cancer samples paraffin-embedded on tissue microarrays (Figure 1(d)). Of the 75 cancerous samples, 40% of tumor samples presented high TNKS expression, but there is no high TNKS expression in paired paracancer samples and normal tissues (Table 1). The clinical data in Table 2 showed that TNKS overexpression was significantly associated with pathological differentiation, tissues types, and tumor size (*P* < 0.05), whereas no association was found with age (*P* > 0.05). These results demonstrated the clinical significance of TNKS serving as a potential molecular target for ovarian cancer patients.

### 3.2. TNKS Is Required for the Growth of Ovarian Cancer Cells

**Figure 2:**
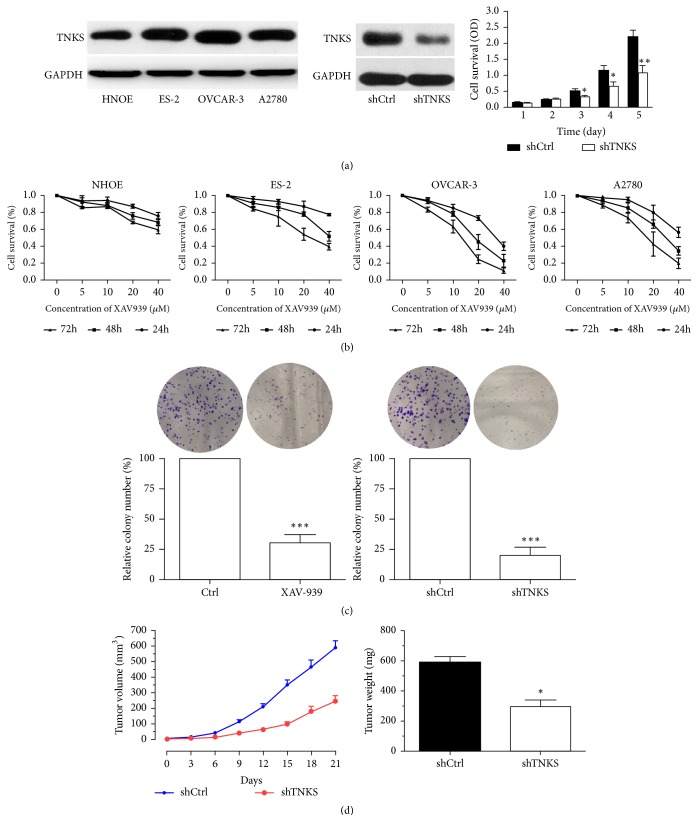
*The Role of TNKS in the Proliferation of Ovarian Cancer Cells*. (a) Western blotting analysis of TNKS protein levels in several ovarian cancer cell lines and normal human ovarian epithelium (NHOE) cells, and the effect of TNKS knockdown on the proliferation of OVCAR-3 cells detected by MTS in shTNKS-OVCAR-3 cells and its control (shCtrl) cells. (n = 3). (b) Effect of XAV939 on the proliferation of different ovarian cell lines detected by MTS (n = 3). There are statistical differences between different time groups or dose groups in ES-2, OVCAR-3, and A2780 cells but not in NHOE cells. (c) Colony formation assays were used to assess the oncogenic potential of TNKS. The relative number of colonies was quantified (n = 3). (d) Tumor growth curve (left) and final weight of shTNKS and shCtrl xenografts in nude mice (n = 6). The shTNKS and shCtrl cells were injected into the flanks of nude mice, tumor diameters measured twice weekly. Data were expressed as the mean ± SD. *∗*,* P* < 0.05; *∗∗*,* P* < 0.01; *∗∗∗*,* P* < 0.01.

To assess the functional role of TNKS in ovarian cancer cells, we knocked down expression of TNKS in OVCAR-3 cells (high TNKS expression) using TNKS-specific short hairpin RNA lentiviruses (shTNKS). Expression of TNKS in shTNKS cells was significantly lower than that of shCtrl cells (Figure 2(a)). In results from MTS assays (Figure 2(a)), the shTNKS-OVCAR-3 cells showed a significantly slower growth rate from 3 days compared with control cells. Similarly, the XAV939 (TNKS-specific inhibitor) significantly inhibited the proliferation of three ovarian cancer cell lines in a dose- and time-dependent manner but showed little effect on the growth rate of nontumoural NHOE cells (Figure 2(b)). Next, data from the colony formation indicated that the ability of ovarian cancer cells to growth in an anchorage-dependent manner was markedly reduced by TNKS inhibition or knockdown (Figure 2(c)). Furthermore, we subcutaneously injected shTNKS-OVCAR-3 cells and its control cells into nude mice to assess the ability of these cells to form tumors in vivo. The results showed that TNKS knockdown led to slower growth rate and lighter tumor weight compared with the control group (Figure 2(d)). Thus, TNKS is required for ovarian cancer cells growth both in vitro and in vivo.

### 3.3. TNKS Decreases Drug Susceptibility of Ovarian Cancer Cells via Regulating Cell Cycle and Apoptosis Progress

**Figure 3:**
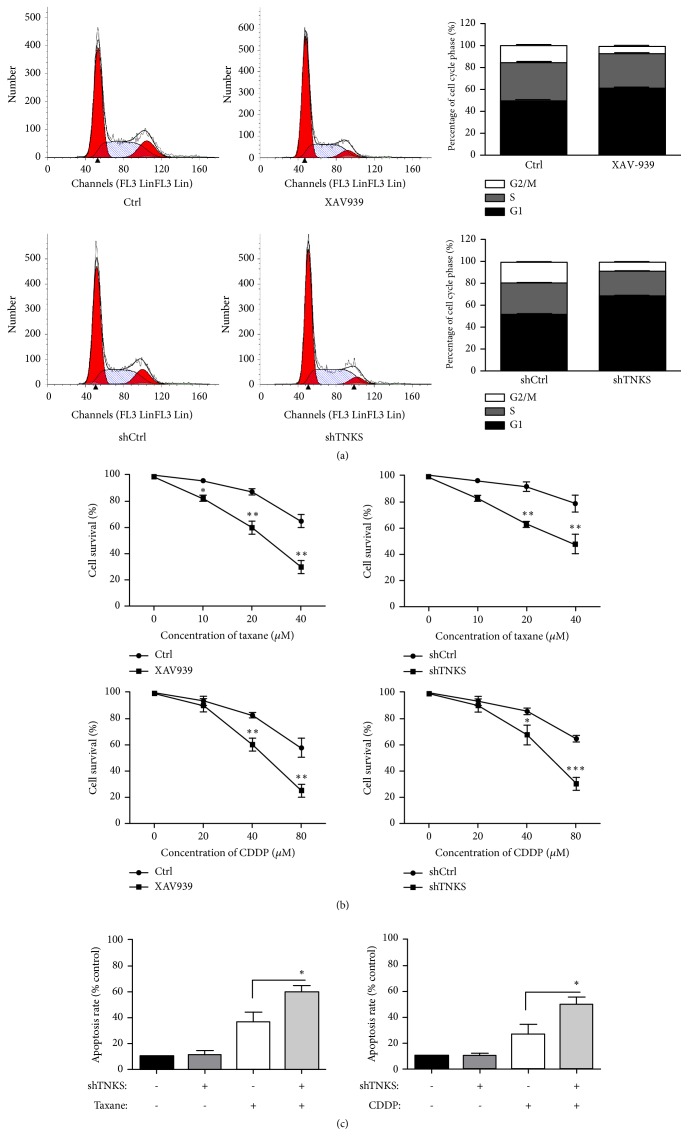
*The Role of TNKS in Cell Cycle and Apoptosis of Ovarian Cancer Cells*. (a) Cell cycle analysis of OVCAR-3 cells after XAV939 treatment or TNKS knockdown performed by flow cytometry. (b) The drug susceptibility of OVCAR-3 cells to taxane and CDDP after TNKS inhibition or knockdown detected by MTS. (c) Cell apoptosis analysis detected by flow cytometry through Annexion V/PI double-staining method. XAV939, 10 *μ*M. n = 3 for each group. Data were expressed as the mean ± SD. *∗*,* P* < 0.05; *∗∗*,* P* < 0.01.

To further investigate the oncogenic potential of TNKS, flow cytometry was performed to assess the cell cycle progress and cell apoptosis. Results from cell cycle analysis showed that TNKS inhibition or knockdown increased the number of cell in G1 phase but decreased the number of cells in S and G2/M phases (Figure 3(a)). In addition, XAV939 and TNKS knockdown significantly enhanced the taxane and cisplatin (CDDP) sensitivity of OVCAR-3 cells (Figure 3(b)). Moreover, a significant increase of apoptosis induced by taxane and CDDP was observed after TNKS knockdown (Figure 3(c)). The biological functions of TNKS in cell cycle and apoptosis might contribute to the drug susceptibility of ovarian cancer cells. Together, these results indicate that TNKS overexpression might contribute to drug resistance of ovarian cancer cells through promoting cell cycle progression and antiapoptosis.

### 3.4. TNKS Promotes the Migratory and Invasive Ability of Ovarian Cancer Cells

**Figure 4:**
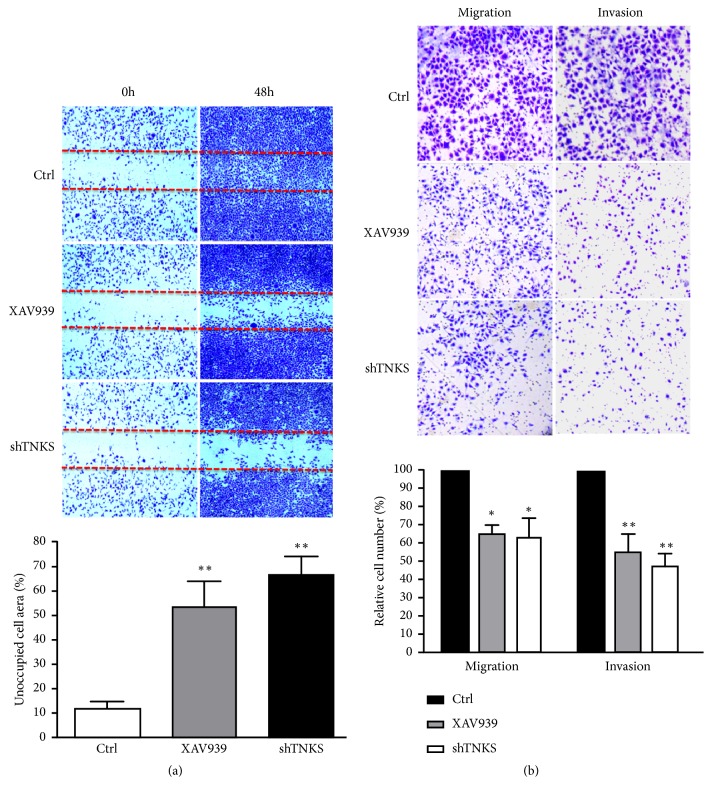
*The Role of TNKS in the Migration and Invasion of Ovarian Cancer Cells*. (a) Cell migration was detected by wound-healing assay. Representative microphotographs at 0 h and 48 h after wounding (top) and a plot of the cell-free area within the wound are shown (bottom). (b) Cell migration (without matrigel) and invasion (with matrigel) were detected by transwell analysis. Representative microphotographs at 48 h after XAV939 treatment or TNKS knockdown (top) and the calculated number from triplicates are shown (bottom). XAV939, 10 *μ*M. Data were expressed as the mean ± SD. *∗*,* P* < 0.05; *∗∗*,* P* < 0.01.

Next the effect of TNKS knockdown on ovarian cancer cells migration and invasion was evaluated by using wound-healing and transwell assays. As shown in Figure 4(a), quantification of the cell-free region in the wound-healing area at 48 h indicated that XAV939 or TNKS knockdown markedly suppressed the migration of OVCAR-3 cells, compared with the control group. In line with the wound-healing assay, results from transwell analysis showed that the migratory and invasive abilities of OVCAR-3 cells were significantly suppressed by TNKS inhibition or knockdown (Figure 4(b)). Hence, these results suggested that promoting metastasis might be one of the oncogenic potentials of TNKS in ovarian cancer.

### 3.5. TNKS Promotes the Warburg Effect through Upregulating PC

**Figure 5:**
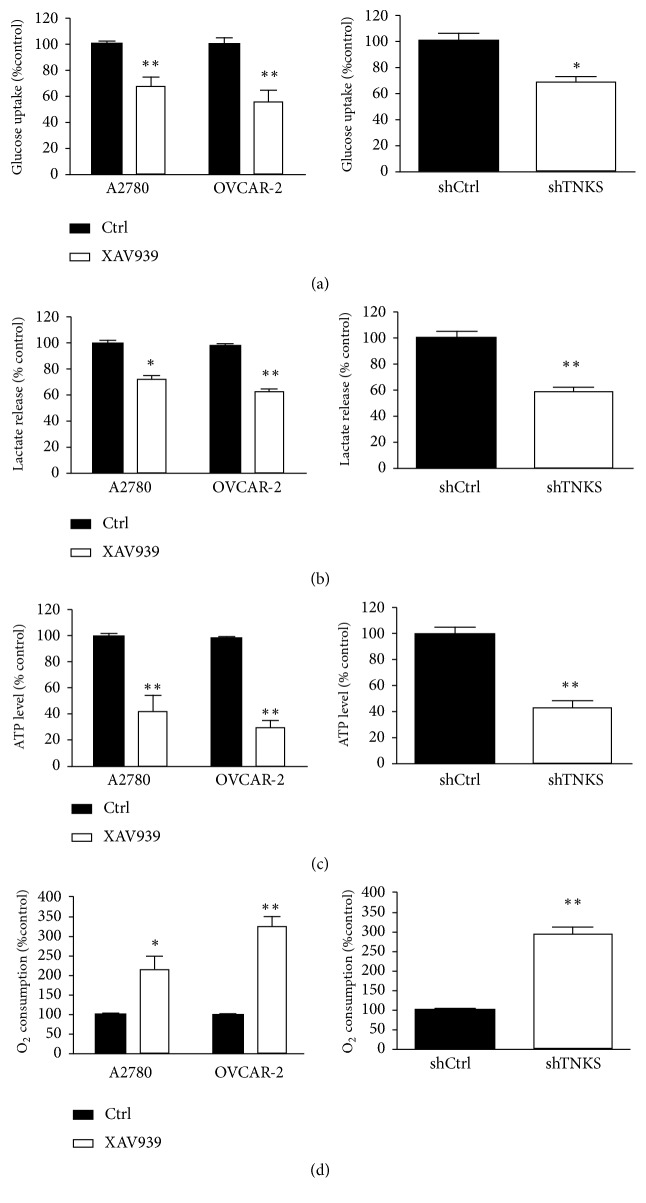
*TNKS is Critical for Glycolytic Activity of Ovarian Cancer Cells*. Cellular glucose uptake (a), lactate release (b), ATP levels (c), and O_2_ consumption rates (d) were measured in A2780 and OVCAR-3 cells after XAV939 treatment for 24 h or TNKS knockdown in the OVCAR-3 cells. XAV939, 10 *μ*M. n = 3 for each group. Data were expressed as the mean ± SD. *∗*,* P* < 0.05; *∗∗*,* P* < 0.01.

**Figure 6:**
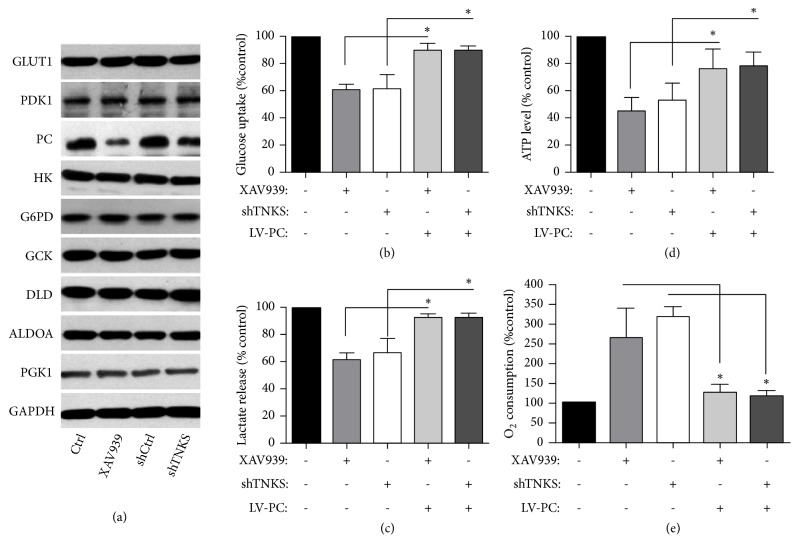
*PC Mediates TNKS-Regulated Aerobic Glycolysis*. (a) Western blotting analysis of aerobic glycolysis associated proteins levels after TNKS inhibition or knockdown. (b-e). Effect of PC overexpression on the cellular glucose uptake (b), lactate release (c), ATP levels (d), and O_2_ consumption rates (e) were measured in TNKS inhibition or TNKS knockdown OVCAR-3 cells. XAV939, 10 *μ*M. n = 3 for each group. Data were expressed as the mean ± SD. *∗*,* P* < 0.05.

To investigate the mechanisms underlying the tumorigenic function of TNKS, we examined whether TNKS1 affected aerobic glycolysis, which is one of the hallmarks of cancer. Compared with control group, TNKS inactivation by XAV939 in OVCAR-3 cells and A2780 cells or TNKS knockdown in OVCAR-3 cells decreased the glucose uptake (Figure 5(a)), lactate excretion (Figure 5(b)), and ATP levels (Figure 5(c)). Moreover, the O_2_ consumption rates were also enhanced (Figure 5(d)). In order to investigate the regulatory mechanism of TNKS in aerobic glycolysis, the enzymes of glucose metabolism were detected using Western blot. As shown in the Figure 6(a), XAV939 and TNKS knockdown reduced the expression level of pyruvate carboxylase (PC) protein, which is a key enzyme involving in glycolytic metabolism. In addition, TNKS inactivation-regulated glucose uptake, lactate excretion, ATP levels, and O_2_ consumption rates (Figures 6(b)–6(e)), suggesting that PC, may mediate the regulation of TNKS in aerobic glycolysis.

### 3.6. TNKS Induces PC through Activation of Wnt/*β*-Catenin/Snail Signaling

**Figure 7:**
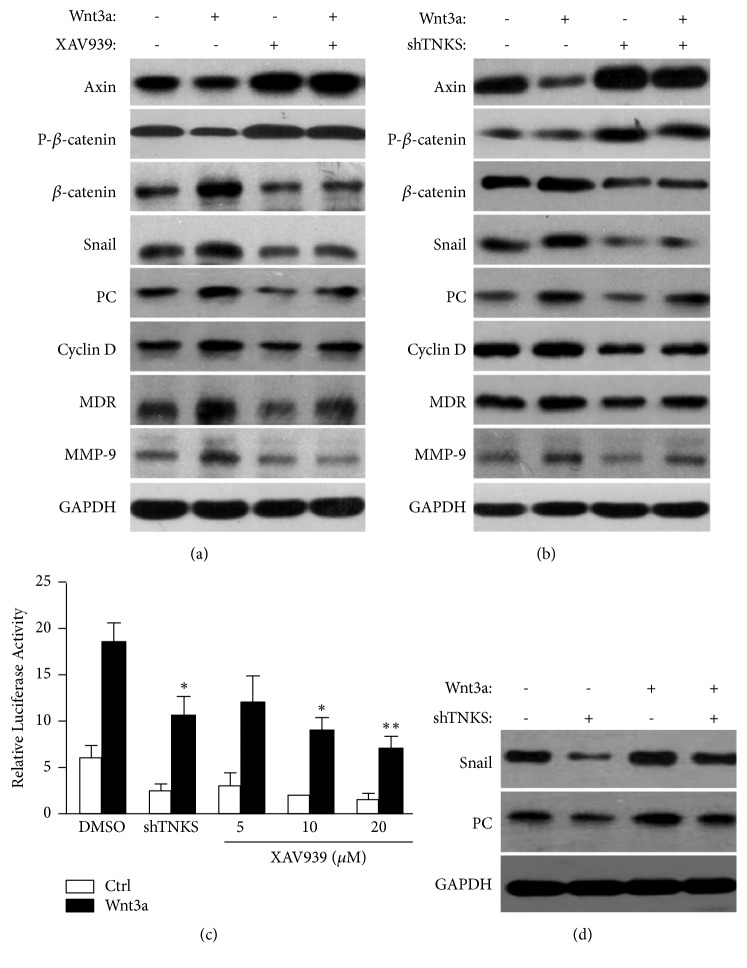
*The Oncogenic Activity of TNKS Exhibits through Wnt/β-Catenin Signaling*. (a) Western blotting analysis of indicated protein levels after XAV939 and (or) Wnt 3a treatment. (b) Western blotting analysis of indicated protein levels after TNKS knockdown and (or) Wnt 3a treatment. (c) Dual-luciferase reporter assay of *β*-catenin/T-cell factor-responsive luciferase activity after XAV939 treatment or TNKS knockdown. (d) Western blotting analysis of snail and PC protein levels after sail knockdown and (or) Wnt 3a treatment. XAV939, 10 *μ*M; Wnt 3a, 30 nM. n = 3 for each group. Data were expressed as the mean ± SD. *∗*,* P* < 0.05; *∗∗*,* P* < 0.01.

Previous work has demonstrated that TNKS could positively regulate the Wnt/*β*-catenin signaling, so we hypothesized that TNKS regulate the targets of Wnt/*β*-catenin signaling to elicit its oncogenic activity. As expected (Figures 7(a) and 7(b)), inhibition of TNKS by XAV939 or TNKS knockdown not only rescued the destabilized of AXIN and decreased of *β*-catenin phosphorylation but also retarded the upregulation of snail, PC, Cyclin D1, MDR, and MMP-9 induced by Wnt3a. Simultaneously, dual-luciferase reporter assay showed that XAV939 or TNKS knockdown markedly decreased Topflash activity (Figure 7(c)), indicating that TNKS could activate the Wnt/*β*-catenin signaling. According to previous studies, we further explored whether snail is critical for TNKS to regulate the expression of PC via Wnt/*β*-catenin signaling. As shown in the Figure 7(d), snail knockdown significantly reduced the expression of PC with or without wnt3a, indicating a role of snail in regulating the expression of PC. Taken together, these results suggested that TNKS could induce PC expression through activation of Wnt/*β*-catenin/snail signaling.

### 3.7. Expression of TNKS Is Positively Associated with Snail and PC in Clinical Samples

**Figure 8:**
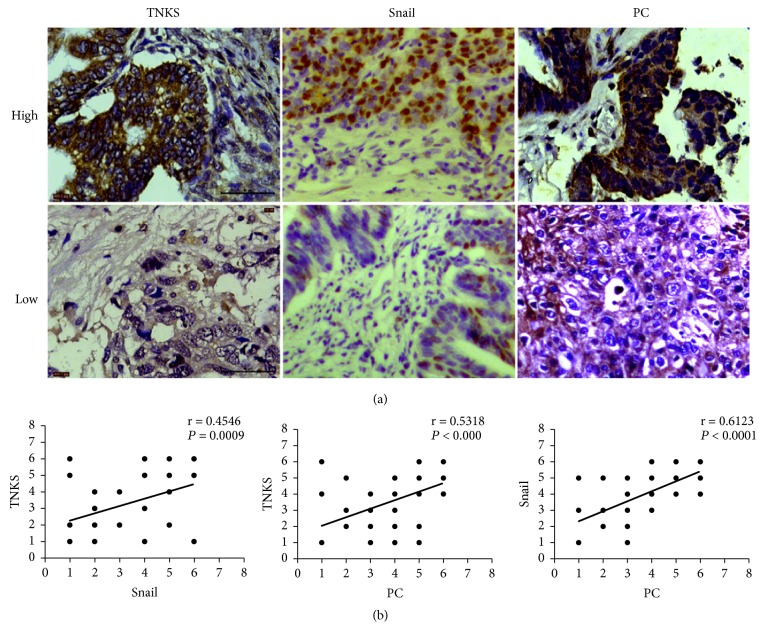
*Clinical Relevance of TNKS, Snail, and PC Proteins*. (a) Representative IHC of TNKS, snail, and PC in ovarian cancer tissues. Scale bar: 50 *μ*M. (b) Pearson's correlation test was performed to analyze the correlations between TNKS and snail, TNKS and PC, and PC and snail protein levels (r and* P* values are shown in the graphs).

To evaluate the correlation between TNKS and Wnt/*β*-catenin/snail signaling and PC in ovarian patients, 75 ovarian cancer samples paraffin-embedded on tissue microarrays were used to detect the expression of TNKS, snail, and PC by IHC (Figure 8(a)). Correlation analyses revealed strong correlations between TNKS and snail (r = 0.4546,* P *< 0.001), TNKS and PC (r = 0.5318,* P *< 0.0001), and snail and PC (r = 0.6123,* P *< 0.0001) (Figure 8(b)). Those above results provide the clinical relevance of TNKS, Wnt/*β*-catenin/snail signaling, and PC.

## 4. Discussion

TNKS shows various biological functions through regulating Wnt/*β*-catenin signaling [11, 18]. Aberrant overexpression of TNKS plays important roles in a several cancers [19, 20]. In this study, we present for the first the role and mechanism of TNKS in the development of ovarian cancer. Our results are in concert with a previous study regarding the aberrant overexpression of TNKS in ovarian cancer and its oncogenic activity exhibited through regulating the targets of Wnt/*β*-catenin signaling.

Previous reports have demonstrated that the frequently hyeractivated Wnt/*β*-catenin signaling contributed to fast proliferation, distal metastasis, and multidrug resistance of ovarian cancer [21, 22]. As a positive regulator of Wnt/*β*-catenin signaling, both of TNKS mRNA and protein levels were overexpressed in human ovarian cancer tissues, compared with the normal fallopian tube epithelium tissues. Epithelial ovarian cancer is a distinct histotype of ovarian cancer that accounts for more than 80% of ovarian cancer patients. In our study, ovarian cancer and its adjacent noncancerous tissues collected from 75 patients who underwent surgery were all fallopian tube epithelium tissues. Furthermore, Inhibition of TNKS by XAV939 resulted in inhibition of cell proliferation, migration and invasion, and the resistance to common chemotherapeutic drugs. Effect of TNKS knockdown by RNA interference achieved similar effects as pharmacological inhibition of TNKS. Mechanistically, those antitumor effects caused by TNKS inhibition or knockdown were associated with increased AXIN protein levels, decreased *β*-catenin protein levels, and decreased *β*-catenin/TCF4 transcriptional activity, which resulted in inactivating Wnt/*β*-catenin signaling and then downregulating the expression of Cyclin D, MMP-9, Snail, and MDR, which were closely associated with the development of ovarian cancer [23–26]. Taken together, TNKS could potently facilitate tumorigenesis and metastasis in many respects throughout the progression of ovarian cancer via activation of Wnt/*β*-catenin signaling. Our findings were in line with previous studies that repressing TNKS activity through either genetic or pharmacological approaches antagonized canonical Wnt/*β*-catenin signaling and inhibited the progression of lung cancer [27], hepatocellular cancer [13], colon cancer [28], gastric cancer [14], and breast cancer [10], indicating that TNKS act as an oncogene may be a common event in the development of cancers.

Importantly, in order to investigate the mechanisms underlying the tumorigenic function of TNKS, we found that TNKS inhibition or knockdown suppressed the aerobic glycolysis of ovarian cancer cells by reducing PC through inhibition of Wnt/*β*-catenin/snail signaling. Many cancers including ovarian cancer need glycolysis to provide primary source of energy even under aerobic condition. This phenomenon is commonly known as the Warburg Effect and is marked by an increased glucose uptake and lactate production [29]. Our study found that TNKS inhibition or knockdown in OVCAR-3 cells significantly reduced the cellular glucose uptake, lactate excretion, and cellular ATP levels and enhanced cellular O_2_ consumption rates. Activation of Wnt/*β*-catenin/snail signaling is known to promote the activity of aerobic glycolysis in tumor cells [30]. The mechanisms of Wnt/*β*-catenin signaling participating in the development of tumor cells are complicated and changeable [31]. Evidence indicated that PC could be a key molecule of Wnt/*β*-catenin signaling and regulate aerobic glycolysis of colon cancer [30, 32–34]. Similarly, we found that PC is required for TNKS to promote glycolysis and snail is critical for TNKS to regulate the expression of PC via Wnt/*β*-catenin signaling, suggesting PC may become a new target to involve in TNKS-regulated the development of ovarian cancer.

Taken together, our findings identified TNKS as an oncogenic regulator of ovarian cancer cells proliferation that promote aerobic glycolysis via activation of Wnt/*β*-catenin signaling. The modulation of this molecular process may be a method of inhibiting ovarian cancer cells growth by downregulation of TNK to interfere with cell metabolism. In particular, the PC may become a new target for further inhibitor design to interfere with Wnt/*β*-catenin dependent ovarian cancer progression.

## Figures and Tables

**Table 1:** Expression of TNKS protein in different ovarian specimens.

Tissue type	N	TNKS stain grades				*X* ^2^	*P*
		-	+	++	+++		
Cancer	75	14	31	21	9	26.53	0.0002
Paracancerous	15	9	6	0	0		
Normal	10	8	2	0	0		

**Table 2:** Associations between TNKS and clinical features of ovarian cancer.

Features			TNKS			
		N	Low	High	*X* ^2^	*P*
Age	<60	51	30	21	0.0919	0.7618
	≥60	24	15	9		

Pathological differentiation	I, II	40	27	13	6.3311	0.0119
	II-III, III	23	8	15		

Pathological tissue	Clear cell adenocarcinoma	6	6	0	13.94	0.0160
	Endometrioid adenocarcinoma	6	4	2		
	Mucinous adenocarcinoma	9	4	5		
	Serous adenocarcinoma	40	22	18		
	Stromal cell cancer	3	0	3		
	Germinoma	6	6	0		

Tumor diameter	d≤10	30	25	5	15.26	0.0005
	10<d<15	18	6	12		
	d≥15	14	5	9		

## Data Availability

The data used to support the findings of this study are available from the corresponding author upon request.
